# Recognition and Diagnosis of *Cryptococcus gattii* Infections in the United States

**DOI:** 10.3201/eid1806.111228

**Published:** 2012-06

**Authors:** Sally Ann Iverson, Tom Chiller, Susan Beekmann, Philip M. Polgreen, Julie Harris

**Affiliations:** Johns Hopkins Bloomberg School of Public Health, Baltimore, Maryland, USA (S.A. Iverson);; Centers for Disease Control and Prevention, Atlanta, Georgia, USA (T. Chiller, J. Harris);; University of Iowa Carver College of Medicine, Iowa City, Iowa, USA (S. Beekmann, P.M. Polgreen)

**Keywords:** Cryptococcus gattii, Cryptococcus neoformans, cryptococcosis, fungal, fungi, physicians, survey, EIN, United States, US Pacific Northwest

**To the Editor:** An outbreak of *Cryptococcus gattii* cryptococcosis has been ongoing in the US Pacific Northwest (PNW) since 1999 (*1**–**3*). In contrast to *C. neoformans* infections, which typically cause meningitis in HIV-infected persons, outbreak-associated *C. gattii* infections occur primarily in persons without HIV and often cause pneumonia (*1**–**3*). Sporadic, nonoutbreak-associated *C. gattii* infections often cause meningitis and have been reported outside the PNW (*1**–**4*). The prevalence of both types of *C. gattii* infection in the United States is unknown because diagnostic practices and awareness vary among physicians.

Some reports indicate that patients with *C. gattii* infections may respond to treatment more slowly and relapse more frequently than patients with *C. neoformans* infections and, thus, may require more aggressive clinical management (*5**–**8*). Therefore, differentiation of *C. gattii* from *C. neoformans* infections may be necessary for optimal patient management. However, cryptococcal infections are often diagnosed by antigen testing, which cannot distinguish between *C. gattii* and *C. neoformans,* and differential agar necessary to distinguish species in culture (*9*) is not uniformly used in clinical laboratories. In addition to possible missed diagnoses caused by the atypical manifestation of outbreak-associated *C. gattii*, outbreak-associated and sporadic *C. gattii* infections in the United States are likely being misdiagnosed as *C. neoformans* infections.

We conducted a survey of US infectious disease physicians to better understand the clinical approach to diagnosing cryptococcal infections, the relative regional frequency of *C. gattii*, and the capacity of clinical laboratories to differentiate cryptococcal species. To survey physicians, we used the Emerging Infections Network (EIN), a sentinel public health surveillance system of infectious disease clinicians that is supported by the Centers for Disease Control and Prevention and sponsored by the Infectious Diseases Society of America (*10*). During February–March 2011, web-based surveys were distributed by email or fax to the 1,342 EIN members, of whom 792 (59%) responded.

Analysis was restricted to 286 (36%) respondents (representing 43 states) who treated a cryptococcosis patient during the past year. We compared answers from respondents in the 4 US census regions (Table; Technical Appendix). Results were analyzed by using SAS version 9.2 (SAS Institute Inc., Cary, NC).

**Table:** Physician responses, by US region, to a survey about cryptococcosis, February–March 2011*

Question and responses	No. (%) responding physicians†				
	Overall, n = 286	Northeast, n = 48	Midwest, n = 63	South, n = 113	West, n = 62
No. patients with cryptococcosis seen during the past year					
1–4	218 (76)	41 (85)	55 (87)	71 (63)	51 (82)
5–8	49 (17)	6 (13)	7 (11)	29 (26)	7 (11)
9–12	12 (4)	1 (2)	1 (2)	8 (7)	3 (5)
>12	7 (2)	0	0	5 (4)	1 (2)
Percentage of patients with cryptococcal pneumonia, with or without meningitis					
0–25	213 (75)	39 (81)	49 (78)	89 (79)	36 (59)
26–50	33 (12)	1 (2)	6 (10)	13 (12)	13 (21)
51–75	8 (3)	1 (2)	1 (2)	4 (4)	2 (3)
76–100	31 (11)	7 (15)	7 (11)	7 (6)	10 (16)
Method used to obtain a diagnosis of cryptococcosis (all that apply)					
Cryptococcal antigen test	272 (95)	48 (100)	58 (92)	110 (97)	56 (90)
Microscopy	95 (33)	16 (33)	13 (21)	42 (37)	24 (39)
Culture	210 (73)	33 (69)	50 (79)	82 (73)	45 (73)
Histopathology	75 (26)	10 (21)	10 (16)	31 (27)	24 (39)
Any combination of tests that does not include culture	76 (27)	15 (31)	13 (21)	31 (27)	17 (27)
Clinical laboratory routinely or on request can differentiate *Cryptococcus neoformans* from *C. gattii*‡	131 (66)	20 (67)	28 (68)	48 (64)	35 (66)
Percentage of cryptococcal infection cases in HIV-uninfected patients					
0–25	154 (54)	32 (68)	26 (41)	70 (62)	26 (44)
26–50	48 (17)	5 (11)	15 (24)	16 (14)	12 (20)
51–75	32 (11)	3 (6)	9 (14)	11 (10)	9 (15)
76–100	51 (18)	7 (15)	13 (21)	16 (14)	12 (20)
Diagnosed cryptococcal infections in HIV-uninfected patients with no known risk factors for infection during past 5 y	78 (27)	6 (13)	13 (21)	26 (23)	33 (53)
Considers species of *Cryptococcus* as a factor of interest in diagnosis or when treating a patient	179 (63)	22 (46)	36 (57)	71 (63)	50 (81)
Considered *C. gattii* infection as a differential diagnosis for pneumonia in a person from the US Pacific Northwest	153 (54)	19 (40)	29 (46)	63 (56)	42 (68)
Ever treated or consulted on a patient known to have *C. gattii* infection	38 (13)	5 (10)	3 (5)	3 (3)	27 (44)

*The survey was conducted by the Emerging Infections Network among physician members; responses are from providers who had seen any patients with cryptococcosis during the preceding year. Region is defined by the 4 census regions: Northeast (Connecticut, Maine, Massachusetts, New Hampshire, New Jersey, New York, Pennsylvania, Rhode Island, Vermont), Midwest (Indiana, Illinois, Iowa, Kansas, Michigan, Minnesota, Missouri, Nebraska, North Dakota, Ohio, South Dakota, Wisconsin), South (Alabama, Arkansas, Delaware, District of Columbia, Florida, Georgia, Kentucky, Louisiana, Maryland, Mississippi, North Carolina, Oklahoma, South Carolina, Tennessee, Texas, Virginia, West Virginia), West (Alaska, Arizona, California, Colorado, Hawaii, Idaho, Montana, New Mexico, Nevada, Oregon, Washington, Wyoming). †Not all respondents answered all questions. ‡Excludes “don’t know” responses.

The approximate number of reported physician consults for cryptococcosis was similar among respondents from all regions (Table). More respondents from the West (40%), compared with the South (21%), Midwest (22%), and Northeast (19%), reported that >25% of their cryptococcosis patients had pneumonia; this finding may reflect the higher prevalence of outbreak-associated *C. gattii* infections in the West (*1**–**3*). The percentage of respondents who treated cryptococcosis patients without known risk factors for infection (including HIV) during the past 5 years was also higher in the West (53%) compared with other regions (Table).

Most (93%) respondents reported that they were aware of the *C. gattii* outbreak. However, only 63% of respondents consider *Cryptococcus* species a factor of interest during diagnosis or treatment, and 54% would consider *C. gattii* as a differential diagnosis for pneumonia in a patient from the PNW. Although awareness of *C. gattii* appears high, recognition of infection may be delayed when diagnostic plans do not include species identification.

Of the respondents, 94% reported that they most often use the cryptococcal antigen test for diagnosis, although 73% of respondents report that they commonly request a culture. Furthermore, 76 (27%) of respondents report using a combination of tests (cryptococcal antigen, microscopy, histopathology) that does not include culture. Tests that do not differentiate between cryptococcal species represent missed opportunities for diagnosis of *C. gattii* infections. When respondents were asked if their clinical laboratory could differentiate *C. neoformans* from *C. gattii* isolates, 131 (46%) responded “yes, either routinely or when requested”; 68 (24%) responded “no”; 87 (30%) did not know. When we excluded respondents who did not know, only 66% of respondents from the West indicated that their laboratory could differentiate species. This finding is concerning because outbreak-associated *C. gattii* is clearly endemic to the region. A better understanding of which laboratories perform this service and which send specimens to a reference laboratory will help identify where additional capacity is needed.

A lower percentage of respondents from the Northeast (10%), Midwest (5%), and South (3%), compared with those from the West (44%), reported having ever consulted on a case of *C. gattii* infection. This may reflect a low incidence of *C. gattii* infections in these regions, or it may be a result of decreased clinical suspicion for *C. gattii* infections outside the PNW.

Results from this study suggest that although most EIN members are aware of *C. gattii* and the ongoing outbreak in the PNW, missed opportunities for diagnosis still exist. To understand the true incidence of *C. gattii* inside and outside the PNW, vigilance among physicians nationwide is necessary. Clinicians and laboratorians should be aware of the need to obtain specimens for culture and of the need to develop methods to differentiate cryptococcal species. An accurate diagnosis of cryptococcosis cases in the United States will lead to a better understanding of the epidemiology and incidence of *C. gattii* in this country and may result in improved treatment.

## Supplementary Material

Technical Appendix Distribution of survey respondents who had seen any patient(s) with cryptococcosis during the past year and who have ever treated a patient with *Cryptococcus gattii* infection, Emerging Infections Network survey, March–February 2011.
