# Supplementary material for: Recognition and Diagnosis of Cryptococcus gattii Infections in the United States
Source: Emerg Infect Dis. 2012 Jun;18(6):1012–5. doi: 10.3201/eid1806.111228 (PMC3358153; doi:10.3201/eid1806.111228)
Supplement: Technical Appendix — Distribution of survey respondents who had seen any patient(s) with cryptococcosis during the past year and who have ever treated a patient with Cryptococcus gattii infection, Emerging Infections Network survey, March–February 2011. [file 11-1228-Techapp_1p.pdf]

Article DOI: http://dx.doi.org/10.3201/eid1806.111228

Recognition and Diagnosis of *Cryptococcus gattii* Infections in the United States

Technical Appendix

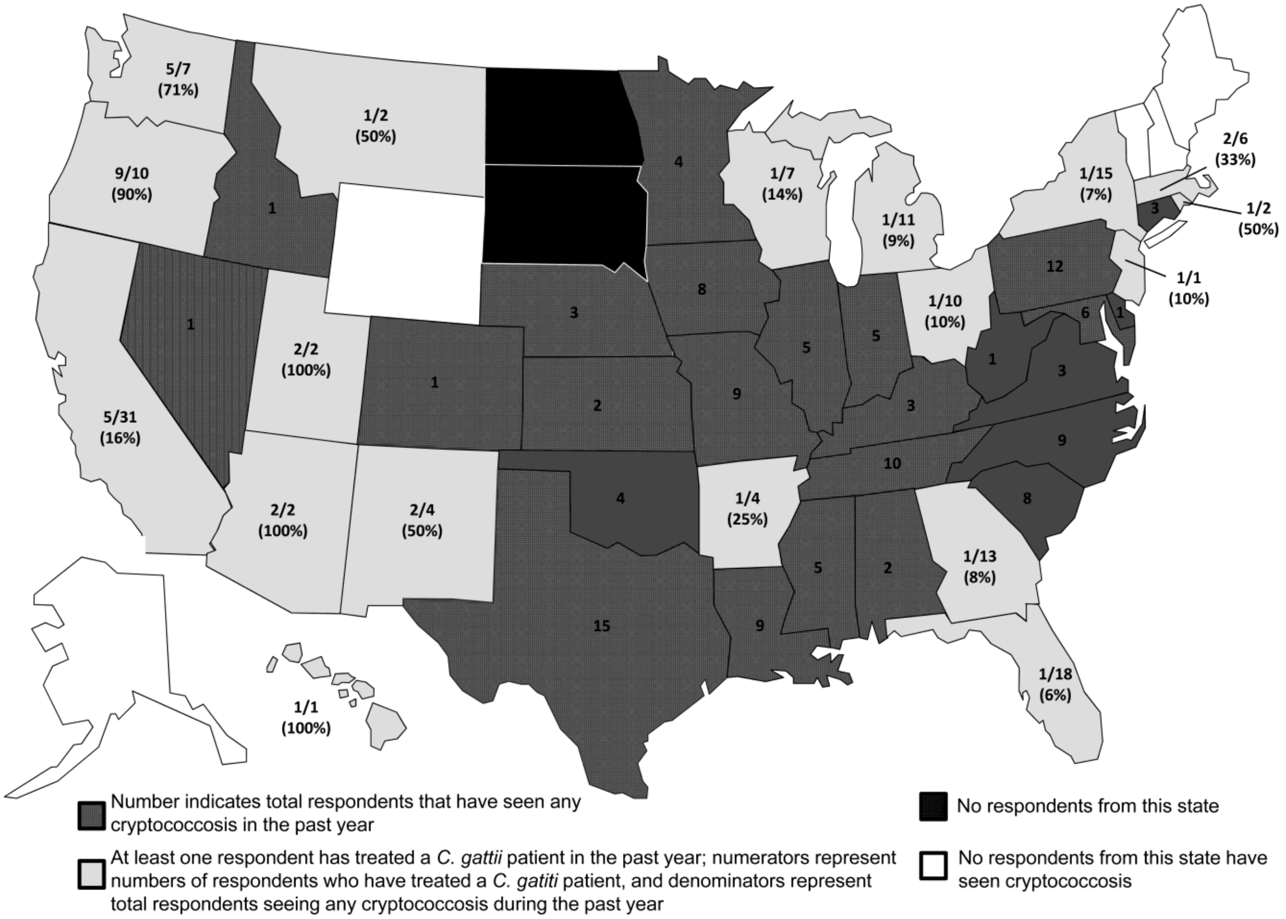

Technical Appendix Figure. Distribution of survey respondents who have seen any patient(s) with cryptococcosis during the past year and who have ever treated a patient with *Cryptococcus gattii* infection, Emerging Infections Network survey, March–February 2011.
